# Acute kidney injury among adult patients with sepsis in a low-income country: clinical patterns and short-term outcomes

**DOI:** 10.1186/1471-2369-16-4

**Published:** 2015-01-16

**Authors:** Peace Bagasha, Frederick Nakwagala, Arthur Kwizera, Emmanuel Ssekasanvu, Robert Kalyesubula

**Affiliations:** Department of Medicine, Internal Medicine and Nephrology, Mulago Hospital and Makerere University, Mulago Hill Road, Kampala, Uganda; Department of Medicine, Internal Medicine and Endocrinology, Mulago Hospital and Makerere University, Mulago Hill Road, Kampala, Uganda; Department of Anaesthesia, Anaesthesia and General Intensive Care, Mulago Hospital and Makerere University, Mulago Hill Road, Kampala, Uganda

**Keywords:** Acute kidney injury, AKIN, Sepsis, Mortality, Resource-limited setting, Africa

## Abstract

**Background:**

Acute kidney injury (AKI) is a common complication of sepsis. We determined the prevalence of AKI among adult patients with sepsis on the medical wards in a low-income country and described their clinical pattern and outcomes at discharge.

**Methods:**

We conducted a cross-sectional study of sepsis-related AKI on the adult medical wards of Mulago National Referral Hospital between January and April 2013. All patients meeting the American College of Chest Physicians (ACP) sepsis criteria were recruited. Demographic, clinical, laboratory and ultrasonography data were recorded and all patients with AKI were followed up to a maximum of 2 weeks. Proportional analysis was carried out and odds ratios with 95% confidence intervals were calculated in the bivariate analysis.

**Results:**

Of 387 patients recruited, 217 (55.6%) were male and the average age was 37 years (range18–90 years). The prevalence of sepsis-related AKI was 16.3%. Age >59 years (*p* = 0.023), a postural drop in systolic blood pressure of >9 mmHg (*p* = 0.015) and a white blood cell count >12,000 cells/mL (*p* = 0.003) were significantly associated with AKI. In-hospital mortality among patients with AKI was 21% (13/63). 59% (20/49) of patients who were discharged alive or were still on the wards after 2 weeks had persistent kidney injury. Acute Kidney Injury Network (AKIN) Stage 3 was significantly associated with persistence of kidney injury (*p* = 0.001). None of the patients requiring dialysis or ICU care received either because of limited access.

**Conclusions:**

The prevalence, morbidity and mortality due to AKI among sepsis patients in Uganda is very high and limited access to dialysis and ICU care is a major factor in poor outcomes for these patients.

## Background

Acute kidney injury (AKI) is a challenging problem in Africa because of the high burden of infectious diseases, late presentation of patients to healthcare facilities and the lack of resources to support patients with established AKI [1]. Sepsis is the most common cause of AKI in critically ill patients (47.5%). Sepsis-related AKI has been associated with a greater severity of illness, an increased risk of death, longer hospital stays compared to non septic AKI yet renal recovery and independence from renal replacement therapy is greater in sepsis-related AKI [2]. Severe sepsis in two Ugandan hospitals was found to have a mortality rate of up to 43%. In-hospital mortality was up to 23.7% and the average length of stay was 6 days for those who survived to discharge [3]. There is little data on the prevalence and outcomes of AKI among patients with sepsis in resource-limited settings (RLS), especially in sub-Saharan Africa although sepsis is highly prevalent because of the high burden of HIV and other infectious diseases [4]. Studies done in developed countries are not comparable to studies in RLS because early patient identification and advanced interventions are routinely available in developed countries and lead to better patient outcomes. Additionally low HIV burden and the advanced age of patient populations with AKI which is common in developed settings also contributes greatly to the potential differences in disease outcome [5].

Our study sought to determine the prevalence of AKI among adult patients with sepsis on the medical wards in the largest hospital in Uganda and to describe their clinical patterns and discharge outcomes.

## Methods

### Patient recruitment and assessment

We carried out a cross-sectional observational study of all patients fitting the ACP sepsis criteria in the emergency department and adult medical wards of Mulago National Referral Hospital in Kampala, Uganda. The hospital provides primary, secondary, and tertiary care services for patients from surrounding health centers as well as from other regional and non-regional referral hospitals throughout the country. The hospital is poorly resourced and consequently most laboratory tests are not routinely available including renal function tests. We consecutively screened all admitted patients aged 18 years and older, for features of sepsis as defined by the American College of Chest Physicians (ACP) [6]. We excluded patients with chronic kidney disease (CKD) as defined by evidence of an abnormal creatinine for >3 months or small kidneys of less than 90 mm on ultrasonography. Patients found to fit the criteria for sepsis with no evidence of CKD were enrolled into the study. The School of Medicine Research and Ethics Committee of the School of Medicine, Makerere University College of Health Sciences approved this study. Written informed consent was obtained from all study participants. For patients unable to give consent because of severity of illness the next of kin was identified and informed consent was obtained.

We interviewed participants using a structured questionnaire to obtain information on the socio-demographic characteristics, presenting complaints, history of prior treatment, and nephrotoxic drug exposure. For patients with a reduced level of consciousness or unable to answer questions because of severe illness answers were sought from the next of kin. We also entered the patient’s working diagnosis while on the ward. Study physicians examined all participants to assess the hydration status and level of consciousness. Height, weight, temperature, blood pressure, respiratory and pulse rates were also obtained. Blood and urine samples were obtained for laboratory testing and 24-hour fluid balance monitoring was charted for each participant. All this data was used to describe and differentiate the clinical patterns of patients who had AKI with sepsis from patients who had sepsis without AKI.

### Specimen sampling and examination

We obtained midstream or catheterized urine samples (4 mL in a standard sterile urinary container) for analysis performed within 30 minutes of sample collection. We performed a dipstick analysis (Combur Test M, Roche Diagnostics, Germany) and recorded the amount of urinary blood, protein, and leukocytes. For microscopy, the remaining portion of urine was centrifuged at 5000 rpm for 10 minutes and the sample was examined by microscope under low (×100) and high (×400) power. We assessed the presence and number of leucocytes, red cells and casts. Serum was analyzed for creatinine against a standard reference range of 66–106 μmol/L.

For patients with a raised serum creatinine (defined as 26.5 μmol/L or more above the upper limit of the standard reference range), we repeated the creatinine test within 48 hours of the first test collection to demonstrate rapidly changing values. The highest value of creatinine was used to stage the patient using the Acute Kidney Injury Network (AKIN) into stages 1, 2, or 3 [7].

### Renal ultrasonography

For each participant with an elevated serum creatinine, an ultrasound scan was performed. Normal renal length, based on American and European studies, ranges from 97 to 112 mm in different populations depending on age group, gender, height, weight and ethnic background. Because of a lack of data on kidney sizes of Ugandans, we characterized a kidney length of less than 90 mm as small and diagnostic of chronic kidney disease. We followed all patients with AKI for up to 2 weeks until discharge or death. At discharge, a repeat creatinine was performed to determine if the AKI had resolved or persisted.

### Statistical analysis

The prevalence of AKI was estimated as the proportion of patients who had AKI among the study participants. Logistic regression was used to identify factors associated with AKI by deriving the odds ratio (95% CI) and *p*-value of the respective relationships. In all analyses, a *p*-value of ≤ 0.05 was considered statistically significant.

## Results

### Demographic characteristics

**Figure 1:**
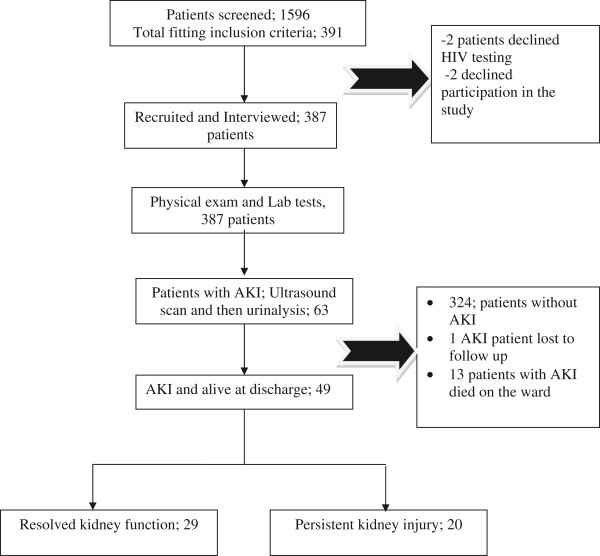
**Patient flow chart.**

We screened 1,596 patients. Of these 391 were were eligible, but 387 patients were enrolled (Figure 1). The mean age was 37 years (range 18–90 years) and 215 (55.6%) were male. Up to 100 (24.8%) participants admitted the use of herbal medication while 12 (3.1%) had exposure to contrast within the past 24 hours (Table 1). Sixty-three out of 387 (16%) patients had AKI. One patient later dropped out.

**Table 1:** **Patient characteristics**

Characteristic	Distribution of participants, Total = 387	
	Number	Percentage
**Age in years**		
**1. 18**–**29**	133	34.37
**2. 30**–**39**	114	29.46
**3. 40**–**49**	70	18.09
**4. 50**–**59**	25	6.46
**5. >59**	45	11.63
**Gender**		
**1. Male**	215	55.56
**2. Female**	172	44.44
**Marital status**		
**1. Never married**	128	33.07
**2. Married/Cohabiting**	225	58.14
**3. Divorced**	18	4.65
**4. Widowed**	16	4.13
**Occupation**		
**1. Unemployed**	117	30.23
**2. Student**	40	10.34
**3. Employed**	166	42.89
**4. Retired**	27	6.98
**5. Peasant**	37	9.56
**Education**		
**1. Never attended**	48	12.40
**2. Primary**	164	42.38
**3. Secondary**	124	32.04
**4. Post secondary**	51	13.18
**Use of herbs**		
**1. Yes**	100	25.84
**2. No**	287	74.16
**Exposure to radio contrast**		
**1. Yes**	12	3.10
**2. No**	375	96.90
**Chronic Diseases**		
**1. None**	338	87.34
**2. Cardiovascular**	8	2.07
**3. Gastrointestinal**	10	2.58
**4. Neurological**	1	0.26
**5. Respiratory**	1	0.26
**6. Hypertension**	16	4.13
**7. Diabetes mellitus**	3	0.78
**8. Malignancy**	2	0.52
**9. Hypertension and diabetes**	8	2.07
**AKIN classification**		
**1. Stage I**	18	29.03
**2. Stage II**	15	24.19
**3. Stage III**	29	46.77

In the bivariable analysis for clinical patterns AKI was less common in patients with weights 45–54 kg (*p* = 0.032) and 55–64 kg (*p* = 0.013); a Glasgow Coma Score less than 14 (*p* = 0.001); a urine output of 0.6-2.4 ml/kg/min (p = 0.001) and more than 2.4 ml/kg/minute (p = 0.036). AKI was more commonly associated with decrease in patients with postural systolic blood pressure greater than 9 mmHg (*p* = 0.015), a white blood cell count greater than 12,000 cells/uL (*p* = 0.003) and in age group >59 years (p = 0.023). At age adjusted analysis however, AKI was more commonly associated with a a urine output of 0.6–2.4 mL/kg (*p* = 0.008) and a white blood cell count greater than 12,000 cells/uL (*p* = 0.003) (Table 2).

**Table 2:** **Age adjusted odds ratios**

Variable	Main outcome of interest				
	No AKI	Presence of AKI	Unadjusted OR (95% CI)	Age adjusted OR (95% CI)	
	Number (%)	Number (%)			P-value
**Age in years**					
**1. 18**–**29**	117 (36.11)	16 (25.40)	Reference:		
**2. 30**–**39**	100 (30.86)	14 (22.22)	1.02 (0.48-2.20)		0.573
**3. 40**–**49**	54 (16.67)	16 (25.40)	2.17 (1.01-4.65)		0.069
**4. 50**–**59**	20 (6.17)	5 (7.94)	1.83 (0.60-5.55)		0.274
**5. >59**	33 (10.19)	12 (19.05)	2.66 (1.15-6.17)		0.023
**Use of herbs**					
**1. Yes**	82 (25.31)	18 (28.57)			
**2. No**	242 (74.69)	45 (71.43)	0.85 (0.46-1.55)	1.05 (0.55-2.03)	0.875
**Postural difference (mmHg)**					
**1. 1**	215 (66.36)	39 (61.90)	Reference	Reference	
**2. 2**–**9**	74 (22.84)	9 (14.29)	0.67 (0.31-1.45)	0.58 (0.25-1.36)	0.213
**3. >9**	35 (10.80)	15 (23.81)	2.36 (1.18-4.73)	1.84 (0.89-3.87)	0.106
**Glascow coma scale**					
**1. ≤14**	51 (15.74)	23 (36.51)			
**2. >14**	273 (84.26)	40 (63.49)	0.32 (0.18-0.59)	0.47 (0.24-0.94)	0.032
**Karnofsky performance score**					
**1. ≤50**	232 (71.83)	50 (79.37)			
**2. >51**	91 (28.17)	13 (20.63)	0.66 (0.34-1.28)	0.94 (0.46-1.92)	0.864
**Urine output (L/24 hrs)**					
**1. ≤0.5**	22 (6.79)	17 (26.98)	Reference	Reference	
**2. 0.6-2.4**	285 (87.96)	43 (68.25)	**0.20 (0.10-0.40)**	0.34 (0.15-0.75)	0.008
**3. >2.4**	17 (5.25)	3 (4.76)	**0.23 (0.06-0.91)**	0.56 (0.14-2.18)	0.401
**Intravenous fluids (Litres)**					
**1. ≤1**	291 (89.81)	55 (87.30)			
**2. >1**	33 (10.19)	8 (12.70)	1.28 (0.56-2.93)	0.67 (0.42-2.73)	0.422
**White blood cell counts**					
**1. ≤12,000 cells/mcL**	277 (85.49)	44 (69.84)			
**2. >12,000 cells/mcL**	47 (14.51)	19 (30.16)	2.54 (1.37-4.73)	2.24 (1.11-4.52)	0.024
**Platelets**					
**1. ≤100,000 cells/mcL**	45 (13.89)	10 (15.87)			
**2. >100,000 cells/mcL**	279 (86.11)	53 (84.13)	0.85 (0.41-1.80)	0.73 (0.34-1.56)	0.415

### Discharge outcomes of patients with AKI

**Figure 2:**
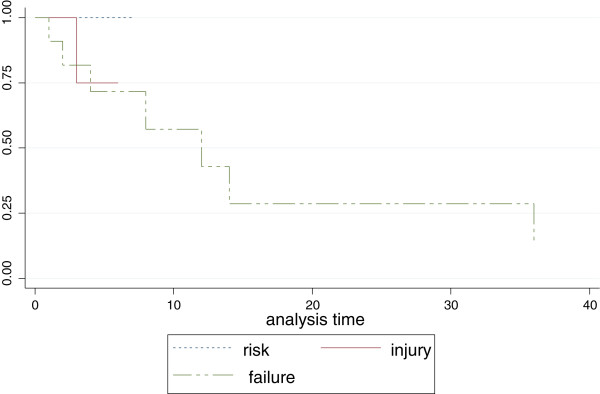
**Kaplan – Meier survival estimates among AKI patients.**

Of 62 patients followed up to discharge or death, 13 patients died and 49 survived to discharge (in-hospital mortality of sepsis-related AKI was 21%). Of the 13 patients who died 12 (92%) had AKIN stage 3 AKI and were eligible for ICU admission and dialysis which were not available. Patients were considered eligible for ICU admission if they had single or multiple organ failure refractory to conservative management, while all patients with renal failure refractory to conservative management were considered eligible for dialysis. Survival estimates showed that patients with stage 3 AKIN had a significantly higher mortality (logrank *p*-value = 0.0154) than those with stage 1 or 2 AKI (Figure 2).

HIV-infected patients with sepsis were more likely to have AKI (59%) and 9/13 of the AKI patients who died were HIV positive with a range of CD4 8–361.

### Comparison among the patients who had kidney injury and survived to discharge

20 of 62 patients were discharged with resolved kidney injury, while 29 had persistent kidney injury at the time of discharge from hospital or from the study (after 2 weeks of follow up). Patients who had AKIN stages 2 (*p* = 0.044) and 3 (*p* = 0.001) were more likely to be discharged with persistent creatinine elevation than those with stage 1 disease. Individuals with enlarged kidneys were more likely to be discharged with persistent kidney injury than those with normal sized kidneys (*p* = 0.045). 46% (29/62) of patients with AKI had AKIN stage 3 and were eligible for dialysis or ICU admission, but none of the patients in the study received either management option.

## Discussion

This study describes the prevalence, clinical characteristics and outcomes of sepsis-related AKI at Mulago national referal hospital in Uganda. It is well known that sepsis in any given setting is a common cause of AKI. In Sub-Saharan Africa the profile of patients with AKI is greatly influenced by the high burden of HIV among other infectious diseases and our study provides further data on AKI in sub-Saharan Africa.

In our study of admissions to the casualty department and medical wards, the prevalence of sepsis-related AKI was 16.3%, which similar to the prevalence of 24% found by Rayner et al. in their study of acute renal failure in community-acquired bacteremia [8]. They also found that persistence of AKI resulted in a higher mortality when compared to bacteremic patients in whom AKI resolved upon treatment of the bacteremia. Sixty-one percent of patients in our study were HIV positive with multiple co-morbidities. HIV infection is associated with pre-existing renal damage from other co-morbid conditions, which may occur independently of sepsis [4, 9].

### Clinical patterns of septic AKI

In the bivariate model we found age over 59 years to be associated with AKI among sepsis patients. This is comparable to the effect of age on AKI in non-RLS [10]. A white blood cell count of >12,000 cells/μL in our study was also significantly related to AKI, which correlates with the severity of AKI pand is consistent with studies done in ICUs [11]. A postural drop in blood pressure of >9 mmHg was also significantly associated with AKI development, which concurs with studies in AKI in the tropics and points to volume depletion and pre-renal azotemia as one of the leading etiologies of AKI [12].

### Outcomes of patients with AKI among the patients admitted with sepsis

Among the patients admitted with AKI, 21% died while in hospital. The majority of patients who died had AKIN stage 3 (92%) which is comparable to other studies in which AKI staging using the AKIN or Risk, Injury, Failure, Loss, End-stage renal disease criteria was a predictor of outcome [10, 13, 14]. Of the patients who were discharged alive, 29 (59%) had persistently elevated creatinine and 20(41%) had resolved kidney injury. Patients who had persistently elevated creatinine were to be followed up for 3 months before they were considered to have CKD but this was not done in our study. Follow-up studies of AKI have shown a high rate of development of end-stage renal disease in elderly patients in particular [11] who had developed AKI in hospital. Patients who had AKIN.

Stage 2 and 3 in our study were more likely to die or have persistently elevated creatinine than resolved kidney injury at discharge. This is similar to other studies that showed poorer patient outcomes with AKIN stage 3 disease compared to stages 1 and 2 [9].

The main eligibility criteria for admission to ICU in Mulago Hospital is single or multiple-organ failure refractory to conservative management. The ICU was inaccessible because of the unavailability of space and financial constraints, while access to dialysis services was limited by the expense. Uganda has one ICU bed for every one million Ugandans (0.1ICU beds/100,000) unlike other developing countries like South Africa (8.9/100,000), Sri-Lanka (1.6/100,000), and the developed like United States (20/100,000) [15]. This contributes greatly to the inaccessibility of ICU to these patients, not forgetting the high costs required for admission.

Uganda has only one national dialysis center, which is located in Mulago National Referral Hospital. It has a capacity of 32 dialysis machines for a population of 34 million Ugandans (0.1 machine/100,000 population) and offers dialysis at subsidized prices, which the majority of patients cannot afford. Surveys to determine dialysis requirements in Uganda or Sub-Saharan Africa are scanty, but in rural America 3.9 residents per 1000 population required dialysis, the majority of whom had end-stage renal disease [16].

### Study limitations

Our study had a number of limitations. For example, we would have liked to do serial renal function tests on all the recruited patients to assess and trend the incidence of AKI during the entire hospital length of stay. Other useful tests would have been CD4 counts and HIV viral load counts for all the patients to assess the impact of HIV on severity of AKI in sepsis and the possible development of HIV associated nephropathy. A point of care measurement of lactate for lactic acidosis, which is a marker of severe sepsis would have also added useful information, but this test are not a part of routine care at Mulago National Referral Hospital.

Additionally, patient follow up for at least 3 months would have provided some information such as outcomes for AKI in low income countries as this would show what percentage of patients go on to develop chronic kidney disease.

## Conclusion

Sepsis related AKI in RLS is more likely to occur in patients >59 years old, with a postural drop in blood pressure and a white blood cell count of over 12,000 cells/mL. High mortality is most probably due to a lack of access to dialysis and ICU services for all patients in the study.
